# Editorial: Stem Cells in Oral Cavity: From Development to Regeneration

**DOI:** 10.3389/fcell.2022.840771

**Published:** 2022-01-20

**Authors:** Takehito Ouchi, Giovanna Orsini, Anne George, Mikihito Kajiya

**Affiliations:** ^1^ Department of Physiology, Tokyo Dental College, Tokyo, Japan; ^2^ Department of Clinical Sciences and Stomatology, Polytechnic University of Marche, Ancona, Italy; ^3^ Brodie Tooth Development Genetics and Regenerative Medicine Research Laboratory, College of Dentistry, University of Illinois at Chicago, Chicago, IL, United States; ^4^ Department of Periodontal Medicine, Graduate School of Biomedical and Health Sciences, Hiroshima University, Hiroshima, Japan

**Keywords:** stem cells, dental, craniofacial, development, regeneration, mesenchymal stem/stromal cells, neural crest cells, skeletal stem cells

Craniofacial stem/progenitor cells are central research topics in the dental field (Yu et al., 2021; Fan et al.). A tool for tissue engineering and regenerative therapy has received medical and scientific attention. Several studies confirmed that intraoral mesenchymal stem/stromal cells (MSCs) derived from craniofacial neural crest cells (NCCs) are useful cell sources. The application of such oral tissue stem cells appears promising for the regeneration of periodontal tissue, alveolar bone, and mucosal structures lost due to congenital abnormalities, trauma, and infections (Li et al.; Cui et al.). Cranial NCCs populate the future facial region and produce ectomesenchyme-derived tissues, such as cartilage, bone, dermis, smooth muscle, adipocytes, and so on.

Kaucka et al. (2016) reported a great degree of similarity in clonal dynamics between neural crest–and paraxial mesoderm–derived mesenchyme in the face and branchial arches. Their results support a profound similarity between vertebrate face and limb development and a deep homology between these seemingly unrelated structures.

In the long bone, which is a typical model of skeletal stem cells (SSCs), SSCs are generally defined as self-renewing cells with the trilineage potential to differentiate into osteoblast, chondrocyte, osteoblasts, and marrow stromal cells or adipocytes. Markers for skeletal progenitor cell populations identified in postnatal growing bones are expressed by growth plate chondrocytes and undifferentiated marrow stromal cells, particularly those located immediately below the growth plate. So far, many markers are reported for SSCs, including chondrocyte-specific genes (Mizoguchi and Ono, 2021). On the other hand, in the craniofacial region, Longaker’s group reported that mechanoresponsive stem cells acquire neural crest fate in jaw regeneration by using lineage tracing of NCC/chondrocyte responsible gene, Sox9 (Ransom et al., 2018). Authors developed a dissectible model of mandibular distraction osteogenesis and used this model to show that newly formed bone is clonally derived from skeleton resident stem cells. Cell lineage tracing to clarify determination fates and non-biased single cell RNA sequence (scRNA-seq) is a strong tool in cellular and developmental biology (Matsushita et al., 2020). In calvaria, Farmer et al. used scRNA-seq to reveal cell diversity within mouse coronal sutures. The authors generated a single-cell transcriptome and performed extensive expression validation. They found preosteoblast features between the bone front and the periosteum, ligament-like populations on sutures that persist into adulthood, and chondrogenic-like populations in the dura mater under sutures (Farmer et al., 2021). Holmes reported that *Hhip*, an inhibitor of hedgehog signaling, is required for normal coronal suture development (Holmes et al., 2021). These reports strongly support the importance of our deeper understanding of single cell level transcriptomics which in turn are influenced by microenvironment and signaling pathway.

In the dental mesenchyme, the combination of lineage tracing and scRNA-seq is an especially strong tool to visualize complicated and heterogeneous tissue cell types (Krivanek et al., 2020; Pagella et al., 2021). Zhang et al. reported a global mapping of open chromatin regulatory elements during dentinogenesis and illustrated how cells are regulated via dynamic binding of different transcriptional factor families, resulting in odontoblast terminal differentiation. Dental stem cells are generally characterized by their tissue origins (Mattioli-Belmonte et al., 2015). Functionally, dental pulp stem cells (DPSCs) have the ability to proliferate and give rise to several lineages including odontoblasts (Gronthos et al., 2000). Paduano et al. demonstrated the control for dedifferentiation of DPSCs and reported that the rerouting of cell fate could potentially be used to enhance their osteogenic therapeutic potential under physiological conditions. Cell-cell communication and interaction are pivotal to perform biological roles and activate functional abilities. N-cadherin-mediated cell-cell interactions are involved as important factors in controlling cell fate decisions in MSCs. Deng demonstrated that N-cadherin acted as a negative regulator via controlling *β*-catenin activity in the odontogenic differentiation of DPSCs by pharmacological intervention and gene silencing (Deng et al.). Stem cells from human exfoliated deciduous teeth (SHED) have higher proliferation ability compared with adult DPSCs (Miura et al., 2003). Dental pulp cells exist under hypoxia condition in the tooth. Hypoxia inducible factor-1α (HIF-1α) is well known to mediate adaptive functions to ischemic stress. Han et al. revealed that HIF-1α plays an essential role in post-implantation survival and angio-/vasculogenic properties of SHED by maintaining cellular and mitochondrial reactive oxygen species levels, homeostasis, inducing metabolic adaptations, and vascular endothelial growth factor (VEGF) secretion. Janebodin et al. revealed that VEGF receptor plays an important role in dentin regeneration by gene silencing and *in vivo* studies. Thus, ligand and receptor axis such as growth factor and its receptor, hormone and its receptor are always important in cellular and molecular biology (Lyu et al.). As other dental stem cells, stem cells from root apical papilla (SCAP) (Sonoyama et al., 2006; Driesen et al.) and periodontal ligament stem cells (PDLSCs) (Seo et al., 2004) are well studied. Such dental stem cell exerts its therapeutic effect mainly by the secretion of exosomes via the paracrine mechanism as well. Stem cell derived exosomes have special advantages such as high drug loading potentials, high specificity, low immunogenicity, excellent biocompatibility, readily available, low side effects, and nanoscale size. In addition, exosomes regulate many important biological processes such as cell-cell communication, anti-inflammatory, bone formation, angiogenesis, immunoregulation, neuronal growth, and promotion of tumor cell apoptosis and so on (Mai et al., 2021).

In regard to oral epithelium, the keratinized epithelial cells of the tongue are responsible for squamous cell carcinoma. However, little is known about the mechanisms of tissue maintenance and regeneration of these cells. Ueno’s group revealed that stem cells positive for Bmi1 rapidly entered the cell cycle and regenerated the tongue epithelium after irradiation-induced damage. And the removal of Bmi1-positive stem cells significantly suppressed regeneration (Tanaka et al., 2013). These results suggested that the Bmi1^+^ stem cells are important for tissue maintenance and tongue epithelial regeneration. Stratified squamous epithelial stem cells are generally thought to attach to a non-hierarchical single progenitor cell model. Byrd et al. (2019) demonstrated lineage tracing and genetic label retention assays in order to show that the hard palate epithelium of the oral cavity is unique in exhibiting marked proliferative heterogeneity. They showed that stratified epithelia of the oral cavity display unusual proliferative heterogeneity, particularly in the hard palate region. A slow-cycling population residing in the junctional zone niche self-renews through planar symmetric divisions, responds to masticatory stress, and promotes wound healing. To help understanding tissue-specific pathophysiology in oral mucosa, Williams et al. (2021) provided the single-cell transcriptome atlas of the human oral mucosa in healthy individuals and patients with periodontitis. It revealed the existence of a complex cellular landscape in oral mucosal tissue and identified a population of epithelial and stromal cells with inflammatory signature that promote antibacterial defense and neutrophil recruitment.

In the craniofacial and dental region, multiple kinds of tissue types, including mesenchymal and epithelial cells, interact together. To prove their hierarchy and fate commitment, lineage tracing technology is one of the strong tools (Orsini et al., 2015). Evaluation of protein expression patterns and gene ablation based on non-biased scRNA-seq provide new insights into genetic regulation in stem cells and their development. Major regulatory mechanisms that control the transcriptional networks of stem cells are mediated through various types of transcriptional factors. Posttranscriptional regulation is essential for stem cell maintenance and cell fate determination. Important players in posttranscriptional control include RNA-binding proteins and noncoding RNAs (i.e., miRNAs, piRNAs, and lncRNAs). Modification of the translated protein influences a large variety of dental cell activities that regulate stem cell maintenance and differentiation throughout all mammalian species (Bian et al.; Wang et al.)*.*

Lastly, further studies through multifacet evaluations, by combining functional analysis under physiological and pathological conditions, will definitely strengthen this research field.
